# Supplementary material for: Myeloid Cell Leukemia 1 Small Molecule Inhibitor S63845 Synergizes with Cisplatin in Triple-Negative Breast Cancer
Source: Cancers (Basel). 2023 Sep 8;15(18):4481. doi: 10.3390/cancers15184481 (PMC10526511; doi:10.3390/cancers15184481)
Supplement: Supplementary file 1 [file cancers-15-04481-s001.zip › File S1. The original western blot figures.pdf]

File S1. The original western blot figures.

IB: Cleaved Caspase 3 (CC3)

IB: GAPDH

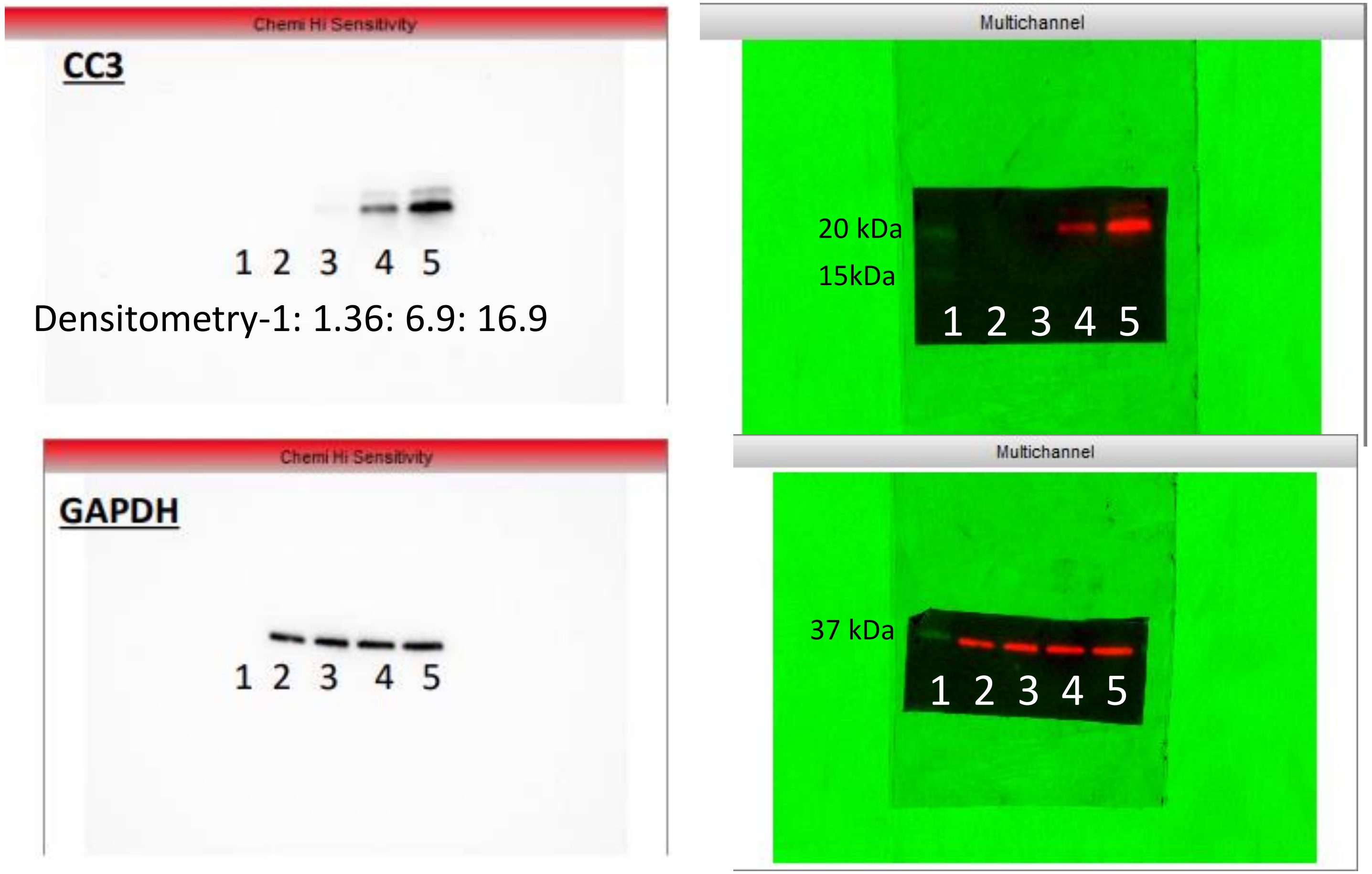

<u>Figure 4E:</u> Lane 1: Ladder. Lane 2: DMSO, Lane 3: 100 nM CSP, Lane 4: 30 nM S63845, Lane 5: Combination

IB: Cleaved Caspase 3 (CC3)

IB: GAPDH

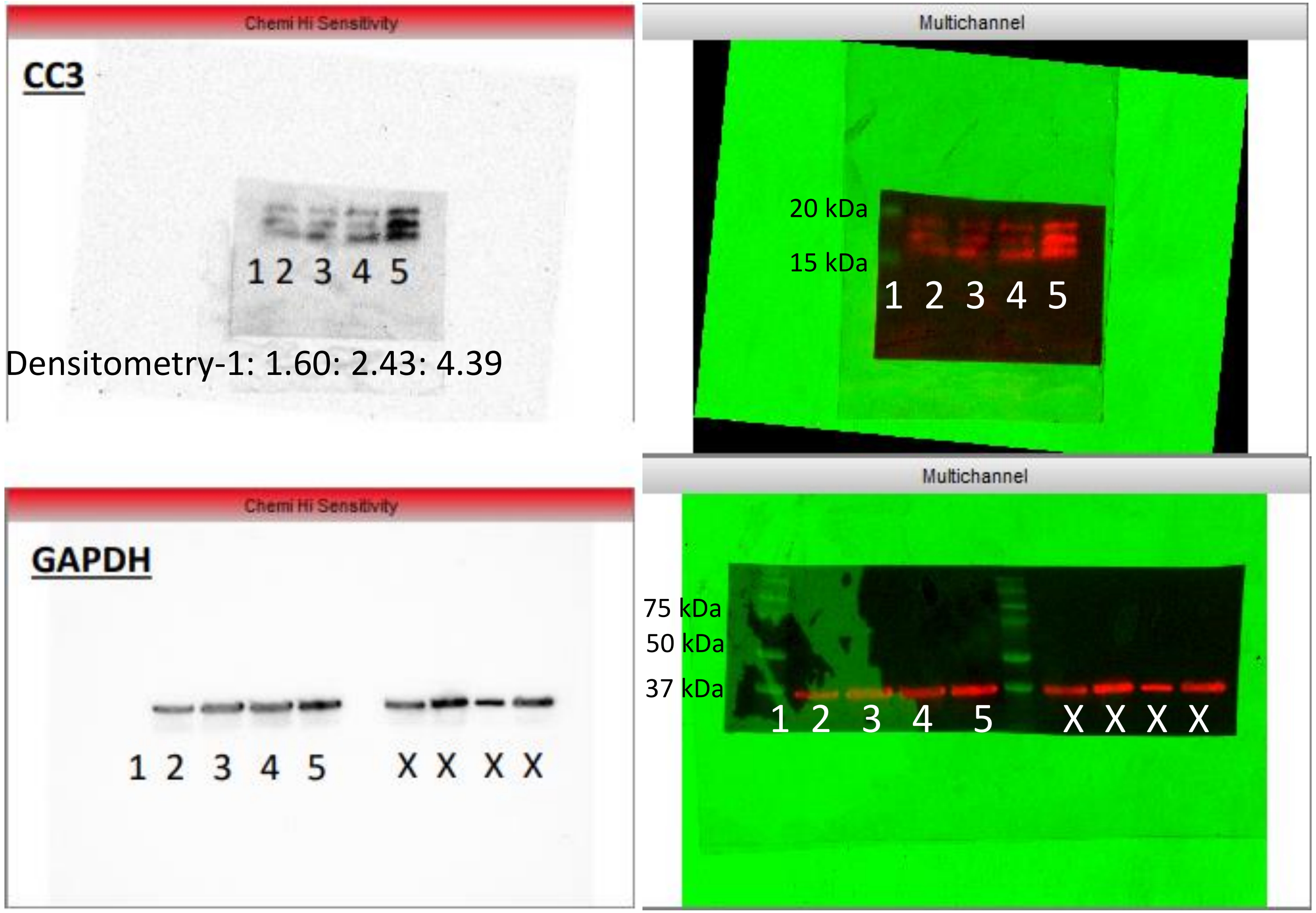

<u>Figure 4F:</u> Lane 1: Ladder. Lane 2: DMSO, Lane 3: 3 uM CSP, Lane 4: 100 nM S63845, Lane 5: Combination, X: Data not in Manuscript

IB: TAp73

IB: MCL1

IB: GAPDH

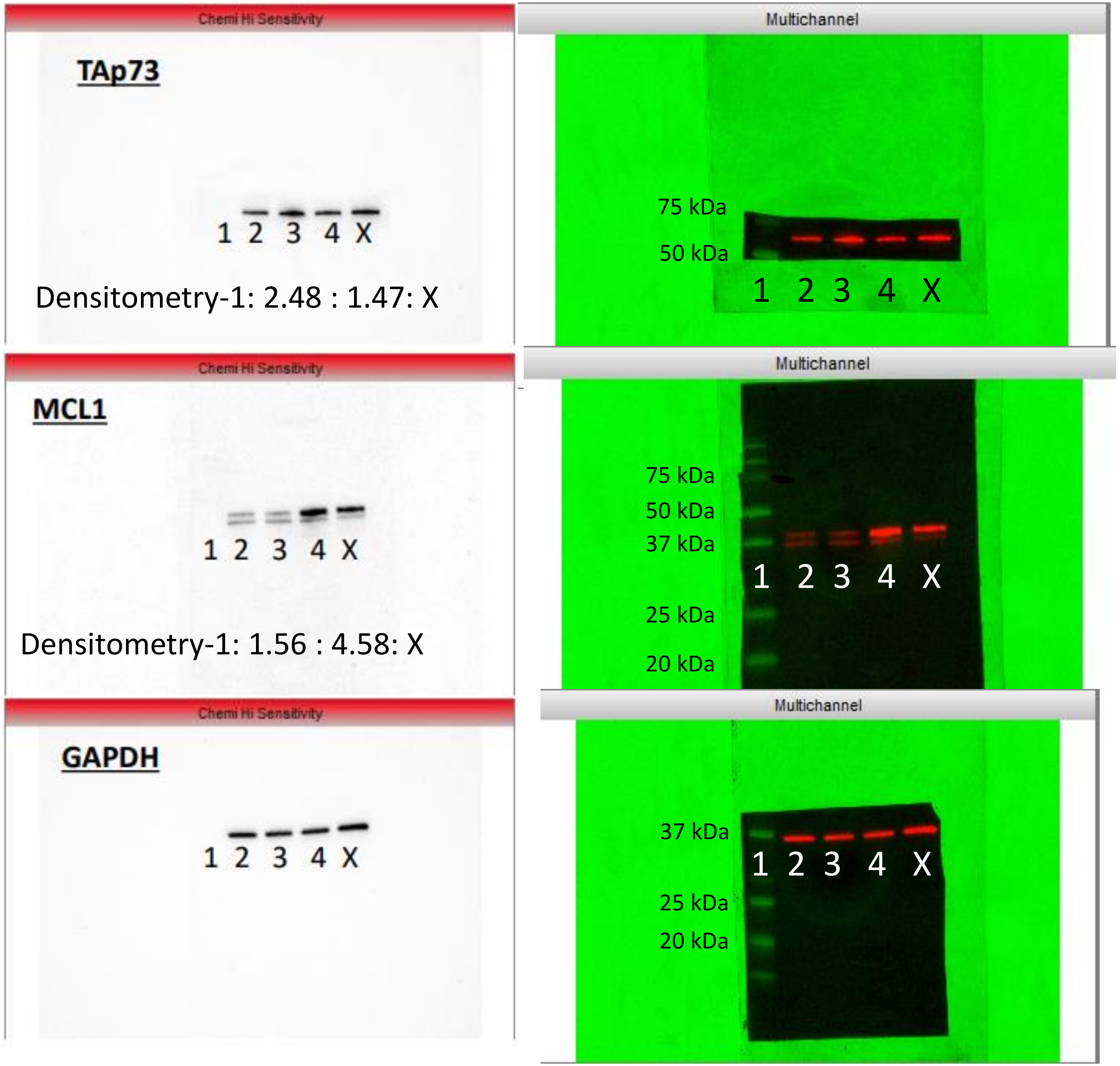

Figure 6C (top panel): Lane 1: Ladder. Lane 2: DMSO, Lane 3: 400 nM CSP, Lane 4: 100 nM S63845, X: Data not included in Manuscript

IB: TAp73

IB: MCL1

IB: GAPDH

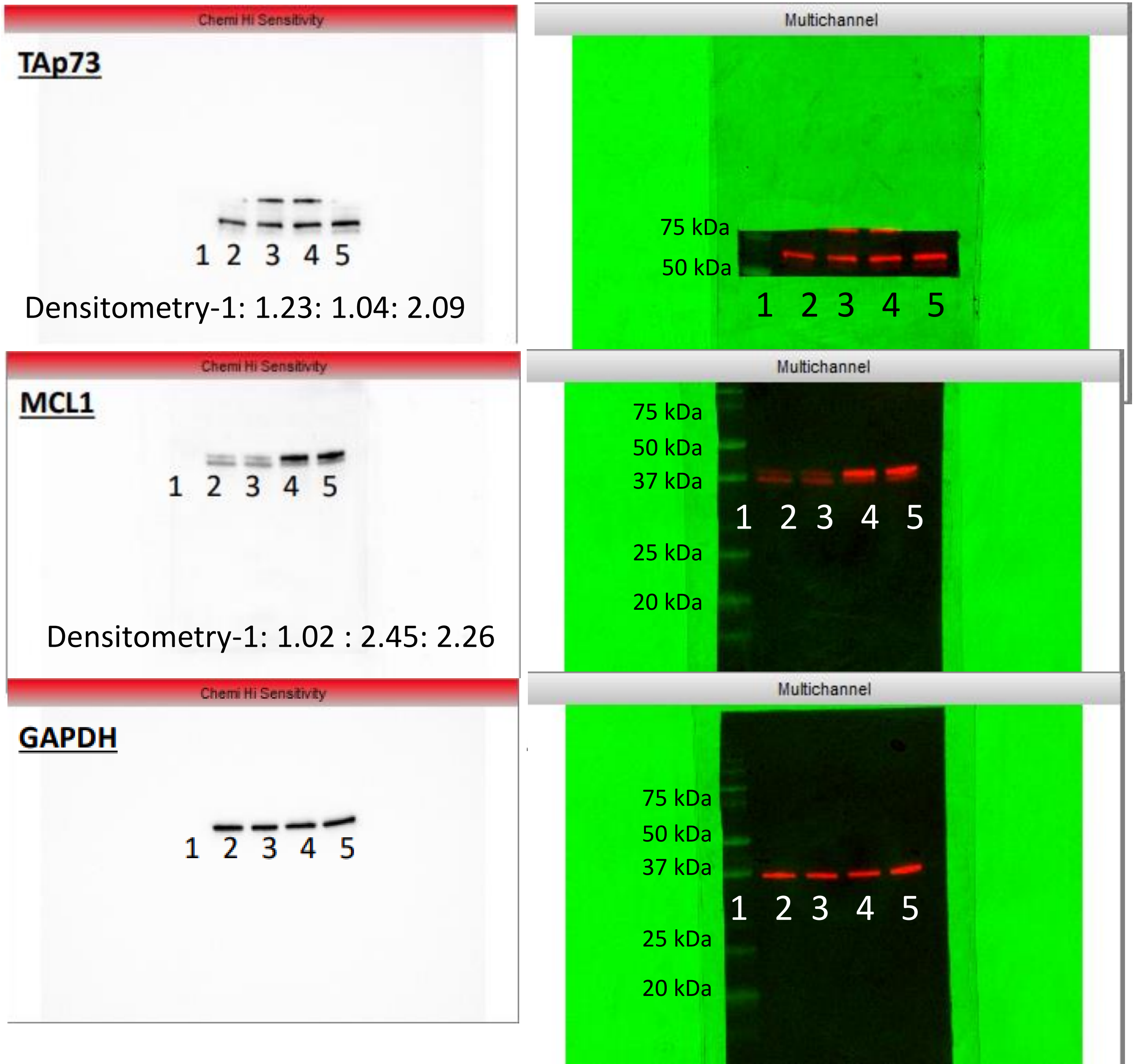

<u>Figure 6C (bottom panel):</u> Lane 1: Ladder. Lane 2: DMSO, Lane 3: 100 nM CSP, Lane 4: 30 nM S63845, 5: Combination

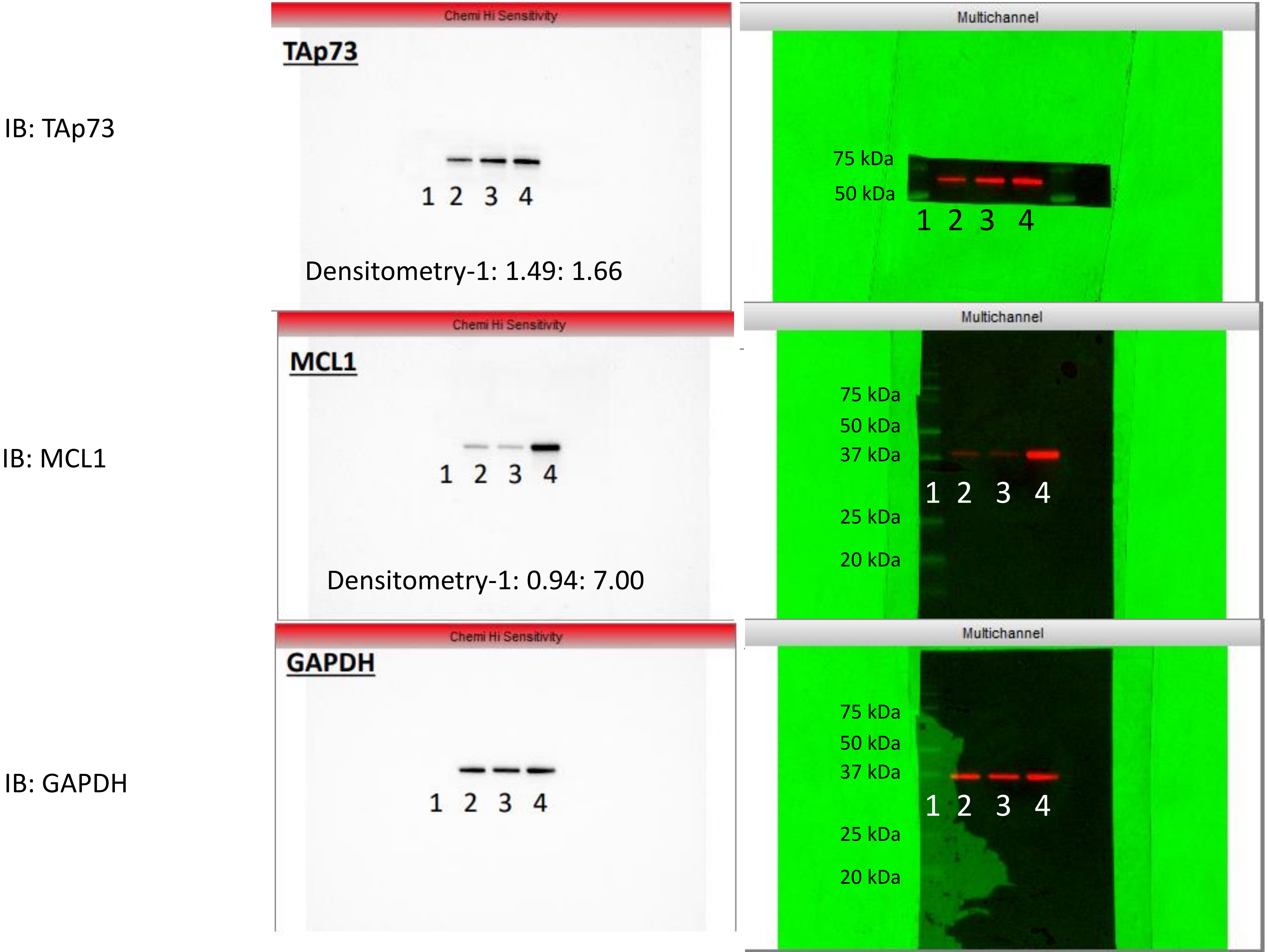

Figure 6D: Lane 1: Ladder. Lane 2: DMSO, Lane 3: 500 nM CSP, Lane 4: 500 nM S63845

IB: MCL1

IB: GAPDH

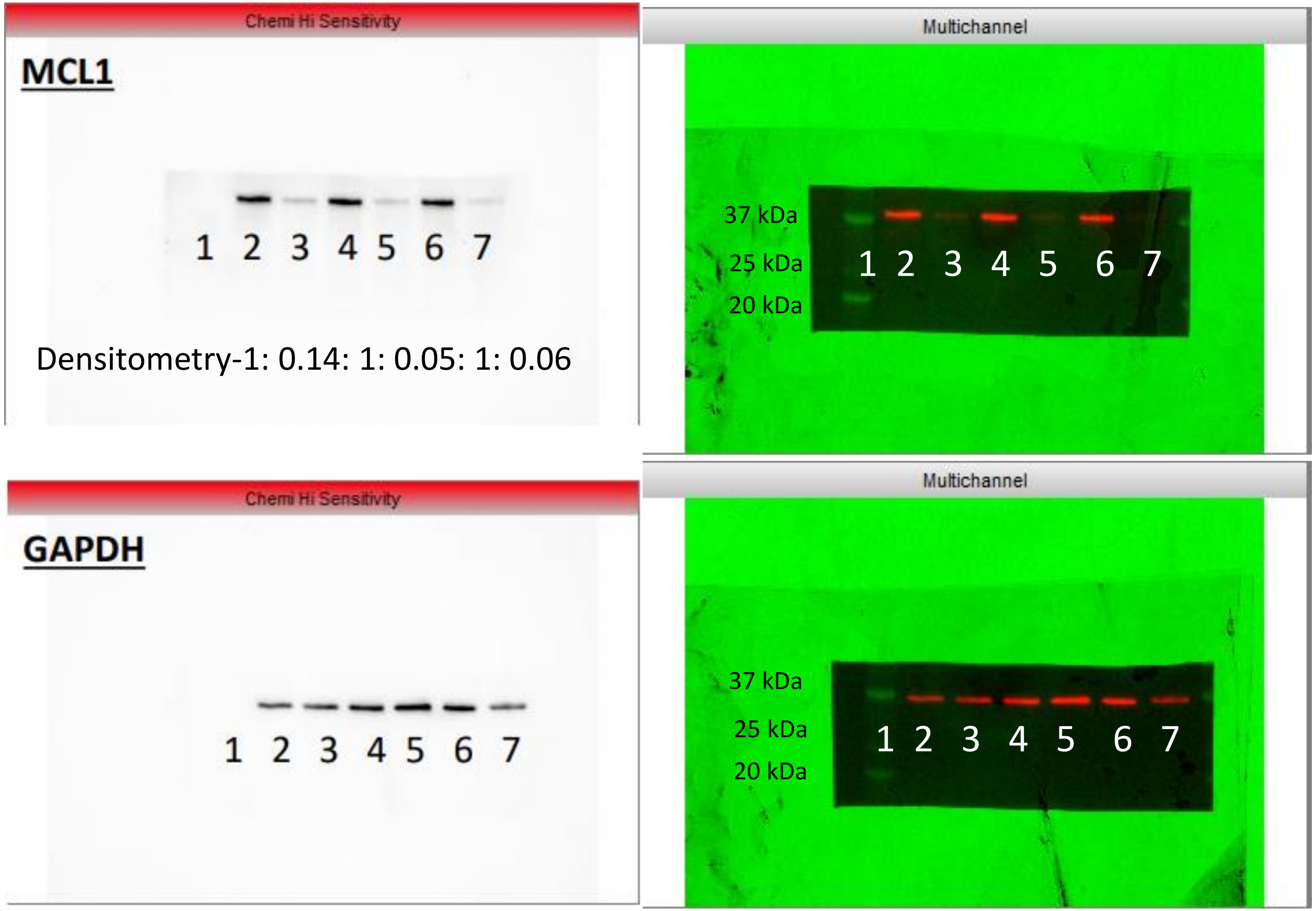

Figure 7A: Lane 1: Ladder, Lane 2: siGFP, Lane 3: siMCL1, Lane 4: siGFP, Lane 5: siMCL1, Lane 6: siGFP, Lane 7: siMCL1

IB: TAp73

IB: GAPDH

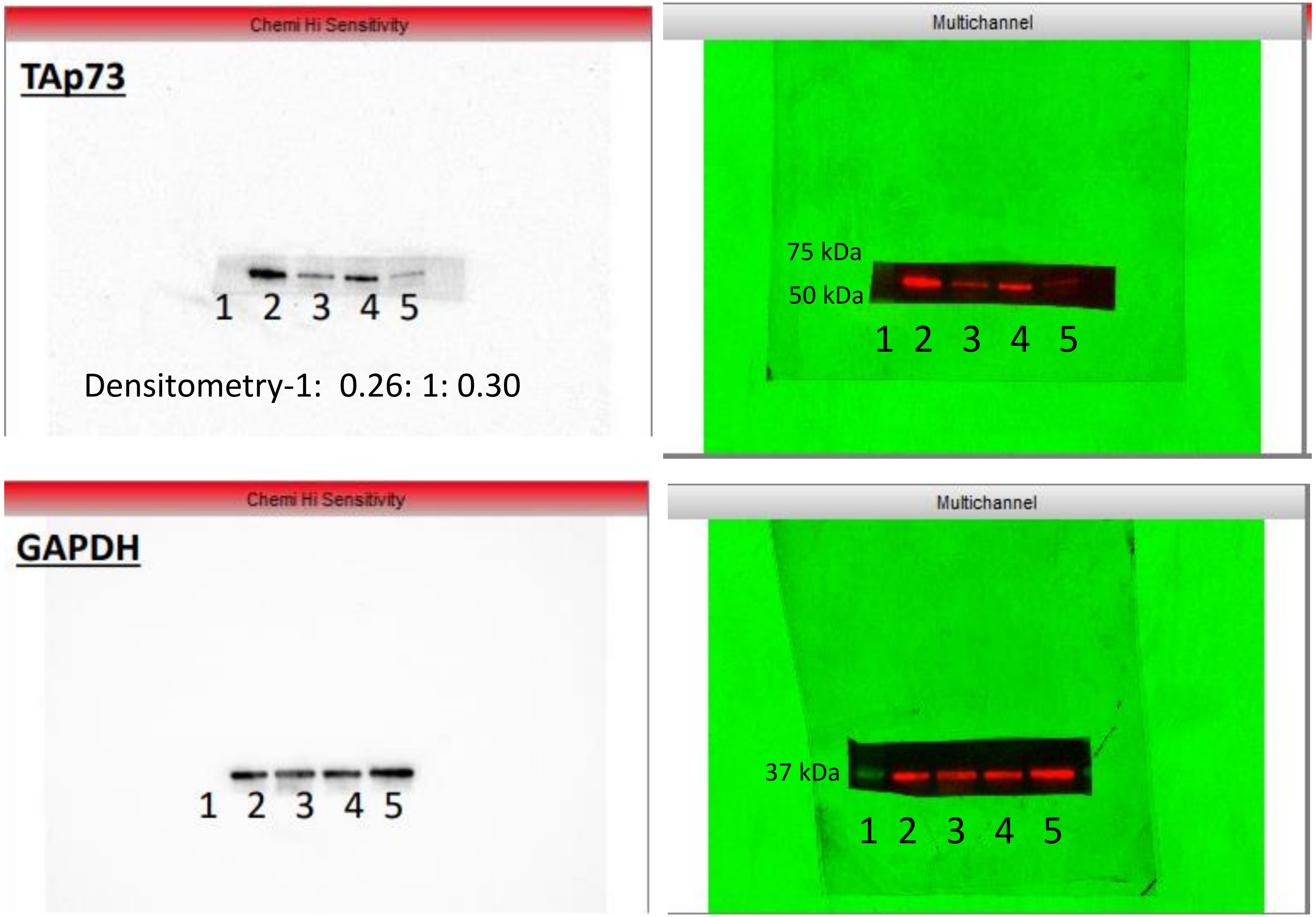

<u>Figure 7D</u>: Lane 1: Ladder, Lane 2: siGFP, Lane 3: siTAp73, Lane 4: siGFP, Lane 5: siTAp73

IB: MCL1

IB: GAPDH

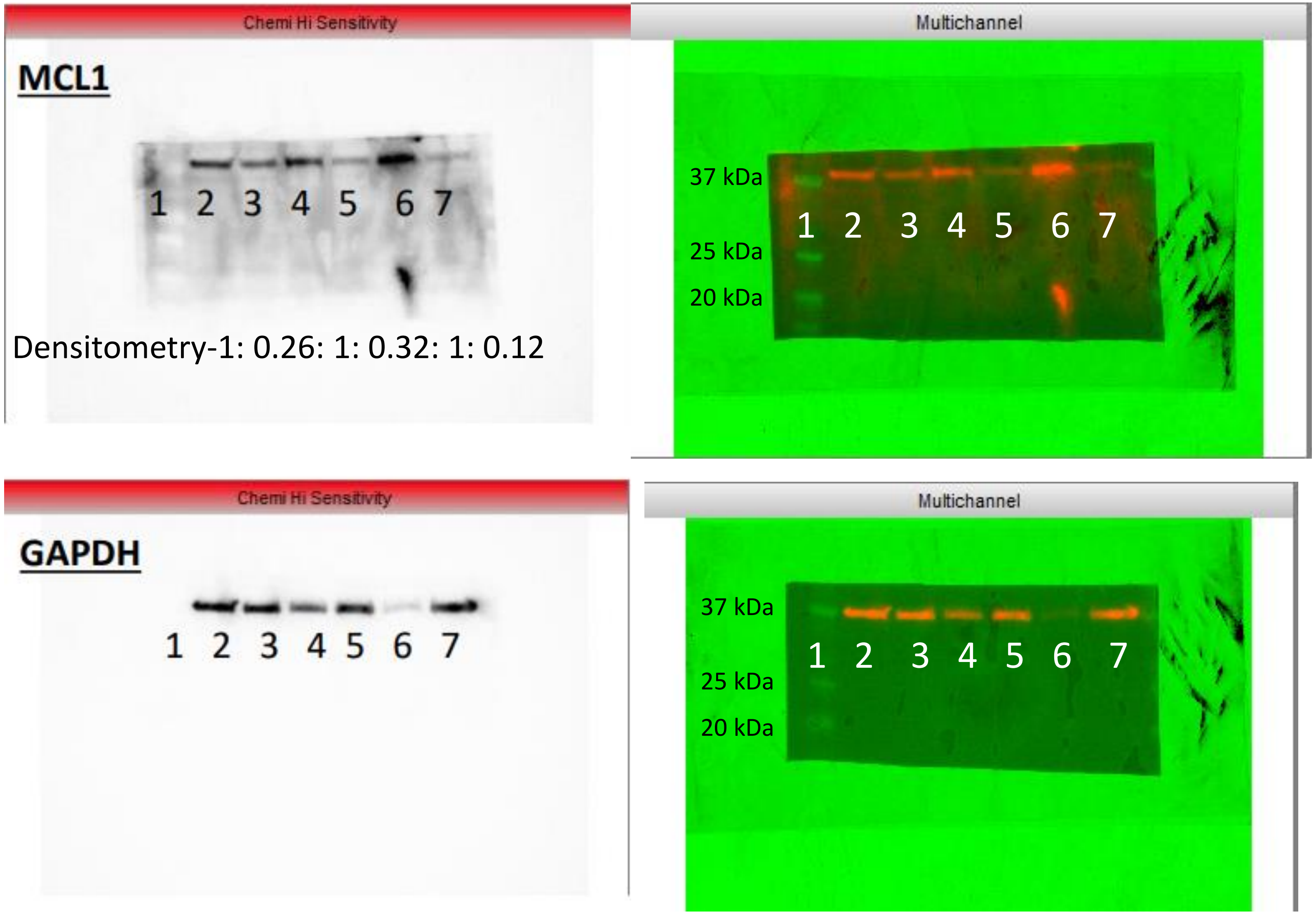

<u>**Supplemental Figure S4A**</u>: Lane 1: Ladder, Lane 2: siGFP, Lane 3: siMCL1 # 2, Lane 4: siGFP, Lane 5: siMCL1 #2, Lane 6: siGFP, Lane 7: siMCL1 #2
