# Supplementary material for: Myeloid Cell Leukemia 1 Small Molecule Inhibitor S63845 Synergizes with Cisplatin in Triple-Negative Breast Cancer
Source: Cancers (Basel). 2023 Sep 8;15(18):4481. doi: 10.3390/cancers15184481 (PMC10526511; doi:10.3390/cancers15184481)
Supplement: Supplementary file 1 [file cancers-15-04481-s001.zip › Supplementary Figures.pdf]

Supplementary Figures:

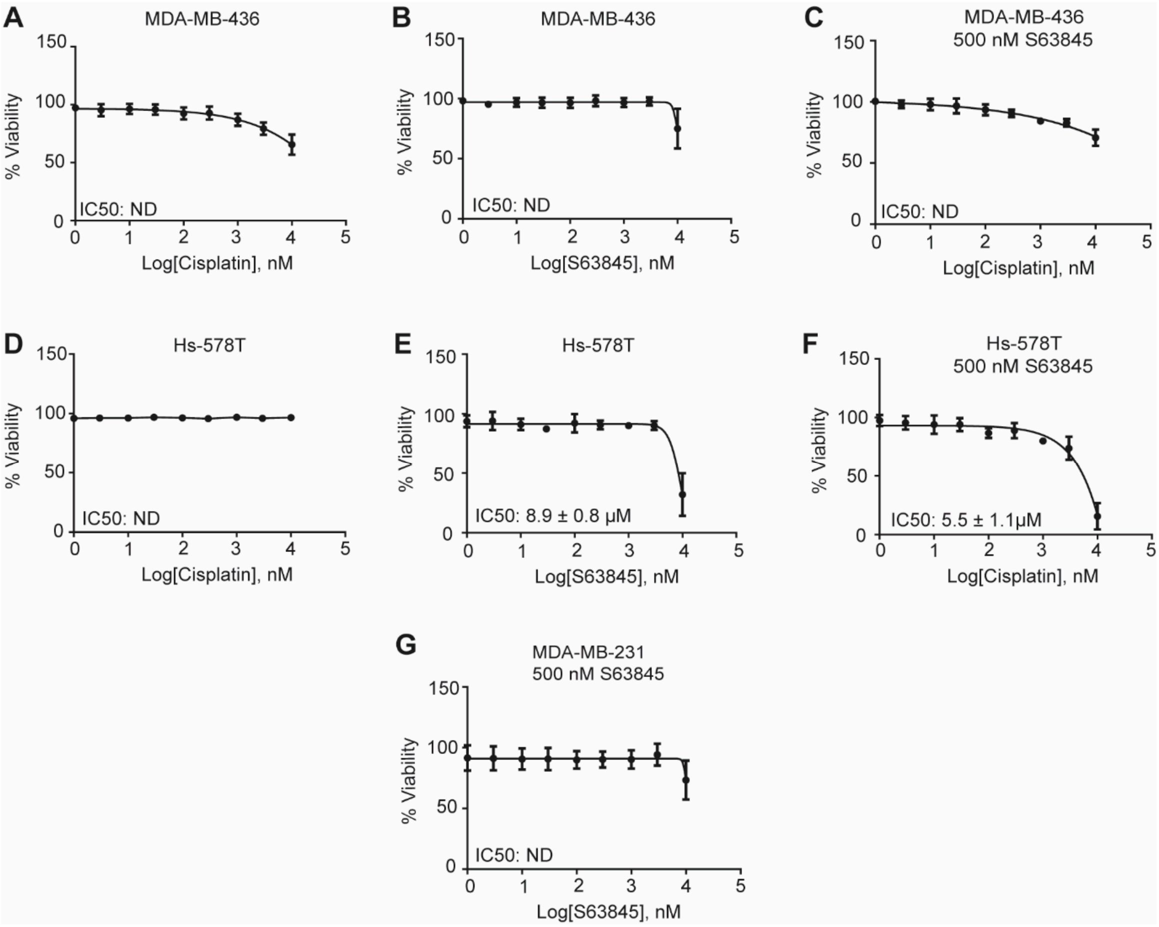

Supplemental Figure S1. MDA-MB-436, Hs-578T, and MDA-MB-231 cells are resistant to cisplatin and S63845. MDA-MB-436 cells were treated with a 9-point dose response curve with (A) cisplatin or (B) S63845 for 72 hours. (C) MDA-MB-436 cells were treated with combination of 500 nM S63845 over a 9-point dose response of cisplatin. Hs-578T cells were treated with a 9-point dose response curve with (D) cisplatin or (E) S63845 for 72 hours. (F) Hs-578T and (G) MDA-MB-231 cells were treated with combination of 500 nM S63845 over a 9-point dose response of cisplatin. Percent viability was analyzed through an MTS assay and IC_{50} values were interpolated using a nonlinear regression on Prism GraphPad. All experiments were performed in biological triplicates and technical replicates.

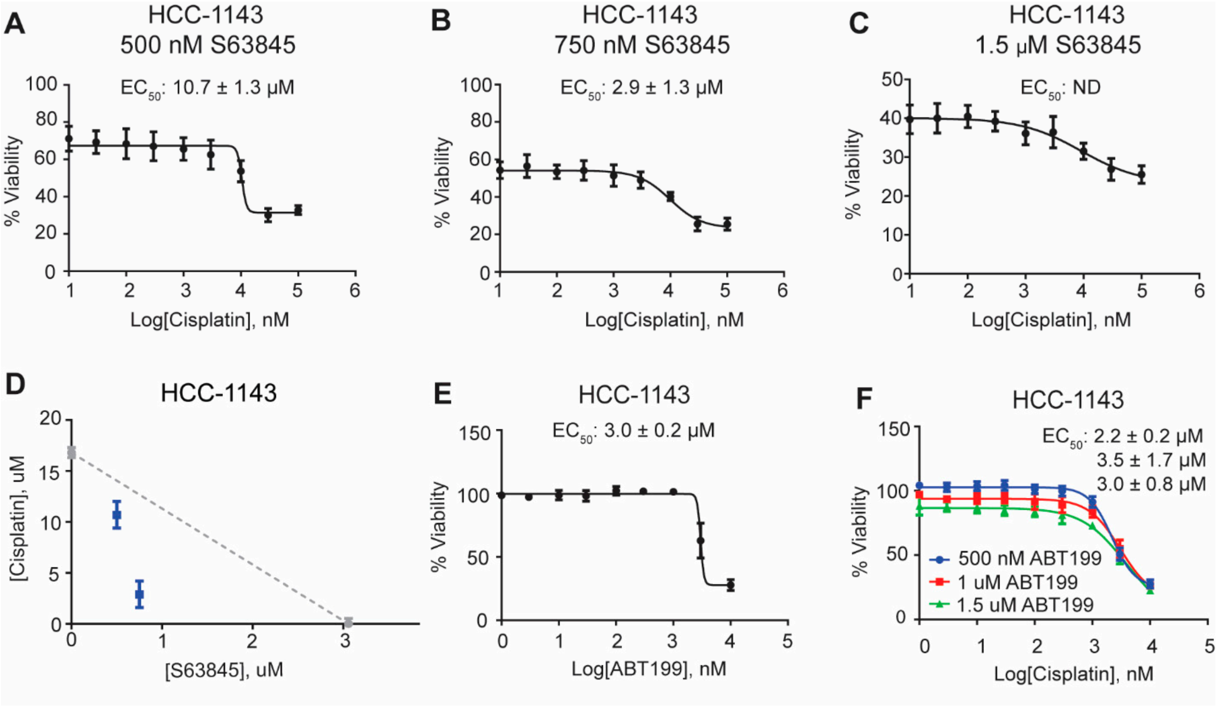

Supplemental Figure S2. Cisplatin and S63845 synergize in HCC-1143 cells. HCC-1143 cells were treated with (A) 500 nM, (B) 750 nM, and (C) 1.5 µM S63845 over a 9-point dose response of cisplatin for 72 h. Isobologram analysis of (D) HCC-1143 cells to represent both single agent (grey) and combination (blue) EC_{50} values. (E) HCC-1143 cells were treated with ABT-199 for 72 h. (F) HCC-1143 combinational studies of various ABT-199 concentrations over a 9-point dose response of cisplatin. Percent viability was analyzed through MTS assay and EC_{50} values were interpolated using a nonlinear regression on Prism GraphPad. All experiments were performed in biological triplicates.

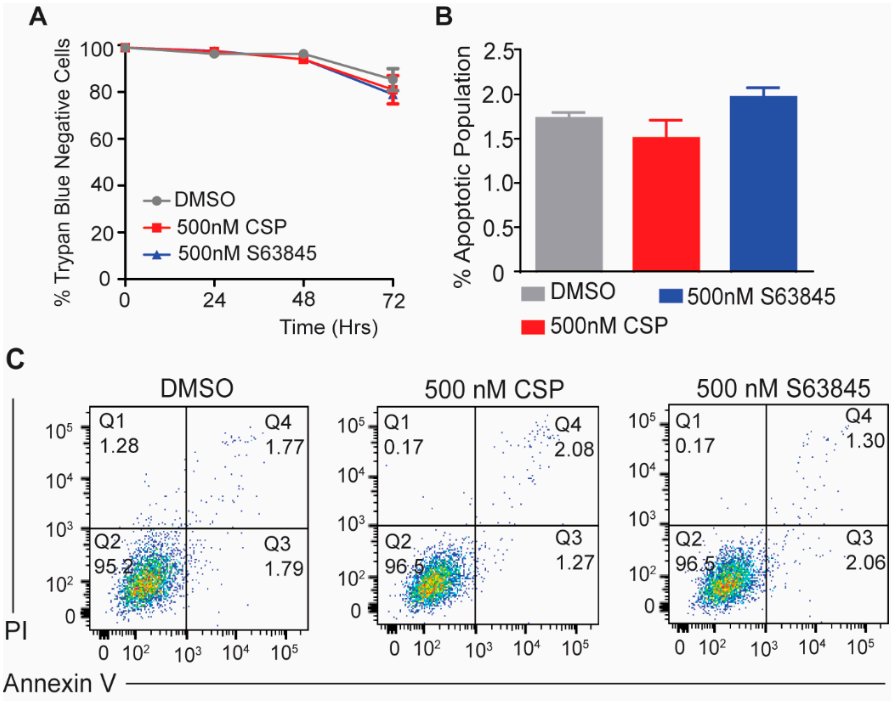

Supplemental Figure S3. Cisplatin and S63845 does not induce apoptosis in MDA-MB-231 cells. (A) Trypan Blue staining indicating percent viability in MDA-MB-231 cells over various treatment groups for 72 h (confluent). (B) Annexin V/PI Q3 quantification of early apoptotic cell population percentage 24 h post treatment. (C) Annexin V/PI staining of MDA-MB-231 were treated with DMSO, 500 nM cisplatin, and 500 nM S63845 for 24 h. Differences between groups were evaluated by a matched one-way ANOVA followed by a Tukey’s post-hoc test, $p < 0.05$. All experiments were performed in biological triplicate.

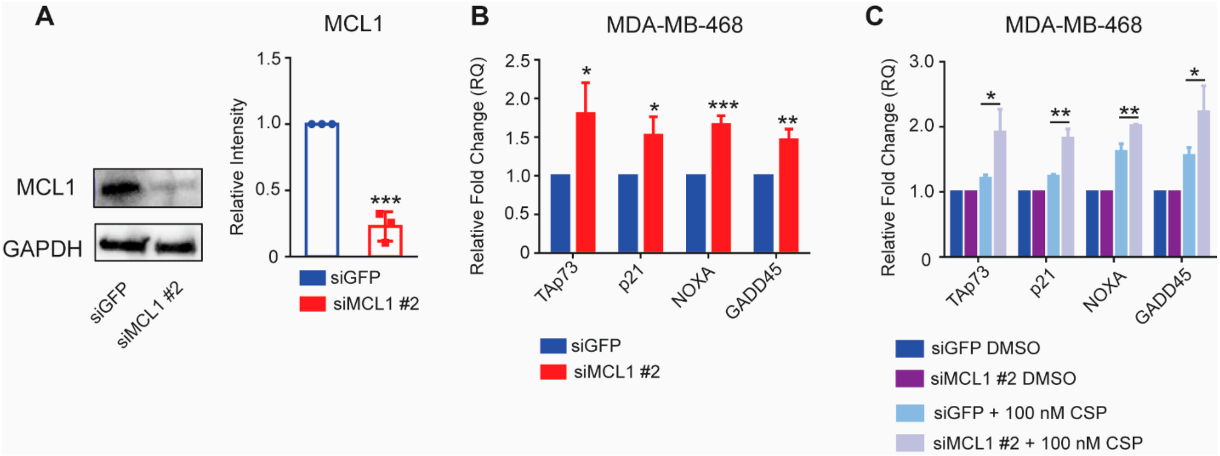

Supplemental Figure S4. siMCL1 increases TAp73 and downstream activation of gene targets. (A) Representative western blot of MDA-MB-468 cells treated with siGFP or siMCL1 #2. RT-qPCR analysis of (B) MDA-MB-468 cells transfected with siGFP or siMCL1 #2 for 24 h. RT-qPCR analysis of (C) MDA-MB-468 cells transfected with siGFP or siMCL1 #2 for 48 h and treated with 100 nM cisplatin for 24 h. Gene expression was normalized to the respective siRNA target treated with DMSO. Differences between groups were evaluated by a matched pair two-way ANOVA followed by a Tukey's post-hoc test, $p < 0.05$. *, $p < 0.05$; **, $p < 0.01$; ***, $p < 0.001$. All experiments were performed in biological triplicate.

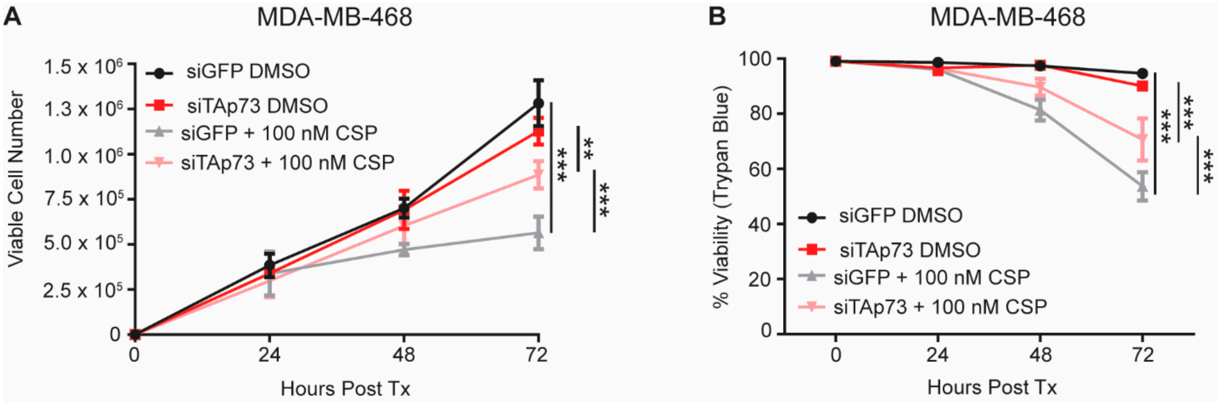

Supplemental Figure S5. TAp73 mediates decreased proliferation and viability in response to cisplatin treatment. (A) MDA-MB-468 cells transfected with either siGFP or siTAp73 and treated with either DMSO or 100 nM cisplatin over 72 h. (B) Matched trypan blue staining of either siGFP or siTAp73 and treated with either DMSO or 100 nM cisplatin treated MDA-MB-468 cells over 72 h. Differences between groups were evaluated by a matched two-way ANOVA followed by a Tukey's post-hoc test, $p < 0.05$. **, $p < 0.01$; ***, $p < 0.001$. All experiments were performed in biological triplicate.
